# Impact of Composition on the Optical Properties of Colloidal Ternary Spinel Oxide Nanocrystals: Spinel Ferrites versus Spinel Gallates

**DOI:** 10.1021/acs.chemmater.5c03447

**Published:** 2026-04-30

**Authors:** Revathy Rajan, Erica P. Craddock, Kathryn E. Knowles

**Affiliations:** Department of Chemistry, 6927 University of Rochester, Rochester, New York 14627, United States

## Abstract

Ternary spinel oxides of formula MB_2_O_4_ are attractive materials for optoelectronic and photocatalytic applications due to their chemical stability and compositional flexibility. Understanding how the compositions of these materials influence their optical properties is crucial for their further development as photoactive materials. Here, we present a quantitative comparison of the absorption spectra of two families of colloidal ternary spinel oxide nanocrystals:metal ferrites (MFe_2_O_4_) and metal gallates (MGa_2_O_4_). We found that, despite the similar crystal structure, shape, and surface ligands of the ternary spinel ferrites and gallates, the optical properties of these materials are significantly different. The ferrites exhibit strong absorption throughout the visible region, with spectra and per iron extinction coefficients that do not change significantly upon changing the identity of the M metal. In contrast, the gallates exhibit significantly larger band gaps and much lower extinction coefficients, with spectra that do depend on the identity of the M metal. We assign the visible absorption observed in ferrites to O 2*p* → Fe 3*d* charge transfer transitions, whereas the visible range transitions observed in some of the gallates arise from intra-atomic *d-d* transitions associated with the M^2+^ cations. We corroborate our findings with band structures calculated by using Hubbard- and Hund-corrected density functional theory computations (DFT+*U+J*).

## Graphical abstract

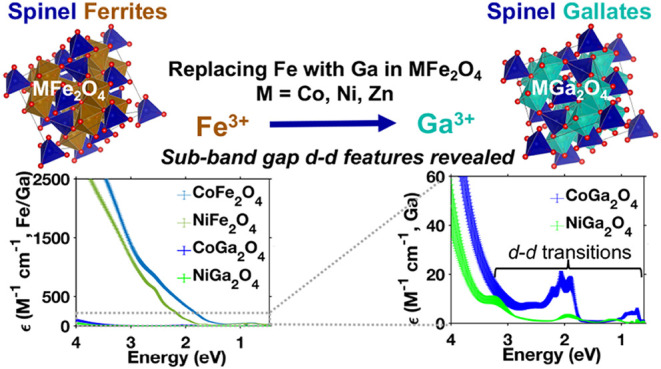

## Introduction

Photodriven reactions are rapidly gaining attention due to their potential to address critical global challenges in energy and environmental sustainability. By harnessing sunlight, the planet’s most abundant and renewable energy source, these reactions can drive chemical transformations such as pollutant degradation or water splitting.^1,2^ Optically active materials with tunable band-edge potentials are increasingly important in meeting the demands of these photoapplications. Spinel oxides of the form MB_2_O_4_ have emerged as a particularly promising class of photocatalysts due to their outstanding chemical and structural flexibility. They are of great interest because of the presence of redox-active metals, band gap energies in the visible region, synthetic tunability, chemical stability, and the scalability of raw materials for application.^3–5^ They crystallize in the cubic space group Fd3̅m and have two distinct metal occupation sites: one with tetrahedral (*T*_d_) coordination geometry, the other with octahedral (O_h_) coordination geometry, which a wide variety of metal cations can occupy.^3^ Because of these two different metal occupation sites, ternary spinel oxides offer the unique ability to assess how the presence of certain metals in specific geometries can be used to tune the band-edge character and optical properties of these materials.

Despite their remarkable compositional tunability and promising photocatalytic performance, the correlation between cation composition and optical properties of ternary spinel oxide nanocrystals remains poorly understood. In this work, we aim to bridge this gap in the literature by directly comparing the optical properties and electronic structures of six different materials spanning two different families of ternary spinel oxide nanocrystals: metal ferrites of the general formula MFe_2_O_4_, and metal gallates of the general formula MGa_2_O_4_, where M = Co, Ni, Zn for each set. We also compare the optical properties of the ternary ferrites and gallates to those of their binary counterparts: Fe_3_O_4_ and γ-Ga_2_O_3_, respectively. Since Fe^3+^ (r = 64.5 pm) and Ga^3+^ (r = 62 pm) have very similar ionic radii,^6^ Ga^3+^ can be substituted for Fe^3+^ without significantly altering the lattice parameter.

We synthesized colloidal nanocrystals of ternary metal ferrites and ternary metal gallates of similar sizes, shapes, and surface ligands, and quantitatively characterized their absorption properties. Despite their similar crystal structures, shapes, and surface ligands, spinel ferrites and gallates displayed markedly different optical features. We demonstrate that when Ga is the B atom in the spinel oxide structure, sub-band gap transitions associated with localized *d-d* transitions of the M cations become more prominent compared to their ferrite analogs. Compared to the corresponding gallates, the ferrites exhibited much larger per-metal extinction coefficients across the UV–visible range. These optical differences arise from intrinsic differences in how the Fe and Ga 3*d* bands contribute to band-edge transitions. Hubbard- and Hund-corrected density functional theory computations (DFT+*U+J*) demonstrate that the spinel ferrite materials contain weakly dispersive conduction band edges arising from the Fe 3*d* orbitals. In contrast, the Ga 3*d* bands are buried deep in the valence band and thus do not contribute to band-edge absorption. In the absence of band-edge states arising from 3*d* bands of the B metal (i.e., Fe), localized intra-atomic *d-d* transitions of the M metal are no longer masked by the onset of a strong continuum absorption arising from O 2*p* → Fe 3*d* transitions. These *d*-*d* transitions are sensitive to the geometry of the transition metal ion and thus qualitatively signify the cation distribution of the spinel oxide. Overall, our results have implications for the development of ternary spinel oxides for optical applications: using transition metals like Fe for the B cation results in strong, broadband absorption, whereas using main group metals like Ga enables narrow, energetically precise absorption features.

## Experimental Methods

### Synthesis of MFe_2_O_4_ and MGa_2_O_4_ Nanocrystals

Into a 25 mL Teflon insert were added 0.7 mmol of iron­(III) acetylacetonate (0.2472 g), 0.35 mmol of M­(II) acetylacetonate (M = Co, Ni, or Zn), 2.7 mmol of oleylamine (0.722 g), 2.7 mmol of oleic acid (0.762 g), and 10 mL of benzyl ether. After stirring for 10 min to make a suspension, the Teflon insert was placed inside a stainless-steel autoclave that was then sealed and heated at 230 °C for 24 h. Three cycles of precipitation with ethanol, followed by centrifugation, were performed to purify the nanoparticles after the autoclave cooled to room temperature overnight. The supernatant was discarded, and the precipitate was dispersed in tetrachloroethylene to obtain colloidal suspensions of the corresponding metal ferrites. The same procedure was followed for the synthesis of Fe_3_O_4_, except for omitting the addition of metal­(II) acetylacetonate. The ferrite nanocrystals all produced yellowish-brown suspensions. The ferrite powders were also attracted to a permanent magnet, indicating that they have a nonzero magnetic moment. For the synthesis of gallates (MGa_2_O_4_ nanocrystals), gallium­(III) acetylacetonate (0.7 mmol, 0.257 g) was used instead of iron­(III) acetylacetonate. γ-Ga_2_O_3_ was synthesized similarly, except for omitting the addition of metal­(II) acetylacetonate. The isolated suspensions of CoGa_2_O_4_ and NiGa_2_O_4_ nanocrystals were blue and mint green in color, respectively, whereas the suspensions of ZnGa_2_O_4_ and γ-Ga_2_O_3_ nanocrystals were colorless. The gallate powders showed no response to the proximity of a permanent magnet.

### Nanocrystal Characterization

#### UV–Vis Absorption Spectroscopy

UV–vis absorption spectra of colloidal dispersions of nanocrystals in tetrachloroethylene were collected in a cuvette with a path length of 1 cm using an Agilent Cary 7000 spectrometer.

#### Transmission Electron Microscopy (TEM)

Samples for TEM analysis were prepared by drop-casting nanocrystal solutions in hexane onto copper grids coated with lacy carbon. All samples were dried overnight at room temperature. All TEM analysis was conducted using an FEI Tecnai F20 transmission electron microscope with a beam energy of 200 kV.

#### Powder X-ray Diffraction

Synthesized nanocrystals were characterized by powder X-ray diffraction (XRD) using a Rigaku XtaLAB Dualflex Synergy-S diffraction system with Mo Kα radiation (λ = 0.71073 Å). The obtained spectra of the nanocrystals were compared to data from the Joint Committee on Powder Diffraction Standards (JCPDS) after converting the 2θ values obtained using the Mo source to 2θ values corresponding to the wavelength of a Cu Kα source (λ = 1.54148 Å).

### Determining the Per-Metal Extinction Coefficient of the Spinel Oxides

#### Atomic Absorption Measurements for Metal Ferrites

The concentrations of the corresponding metals in the metal ferrites (Fe in Fe_3_O_4_, Fe and Zn in ZnFe_2_O_4_, Fe and Ni in NiFe_2_O_4_, and Fe and Co in CoFe_2_O_4_) were obtained from atomic absorption (AA) measurements. A Shimadzu atomic absorption spectrophotometer (AA-7000 series), using element-specific hollow cathode lamps, was used for the measurements. A UV-Vis absorption spectrum of a 0.1 mg/mL colloidal solution of metal ferrite nanocrystals in tetrachloroethylene (TCE) was collected. After drying an adequate amount of the ferrite stock solution under nitrogen, each ferrite sample was digested with 0.5 mL of aqua regia and then diluted to a total volume of 15 mL with Nanopure water prior to AA measurement (see Table S1 in the Supporting Information for more details).

#### ICP-MS Measurements for Metal Gallates

The concentration of the corresponding metals in the metal gallates (Ga in γ-Ga_2_O_3_, Ga and Zn in ZnGa_2_O_4_, Ga and Ni in NiGa_2_O_4_, and Ga and Co in CoGa_2_O_4_) was obtained from inductively coupled plasma mass spectrometry (ICP-MS). A PerkinElmer 2000C ICP-MS instrument, operated in KED mode with a helium flow of 4 mL/min, was used for the analysis. The instrument was operated at a power of 1600 W, with a nebulizer flow of 0.95 mL/min, an auxiliary argon flow of 1.2 mL/min, and a plasma argon flow of 15 L/min. The sample preparation procedure for the metal gallates was similar to that used for the metal ferrites (see Supporting Information for details).

After the metal concentrations in the ferrite and gallate samples were determined from AA and ICP measurements, respectively, the per-metal molar extinction coefficients were calculated using the Beer–Lambert Law and the absorbance values from the corresponding UV-Vis absorption spectra. The uncertainties were obtained as the standard deviation of four independent measurements obtained from two sets of syntheses (two per synthesis). There is negligible batch-to-batch variability in the measured extinction coefficient values.

### Computational Methods

#### DFT*+U+J* Calculations

The electronic structures of the ferrite and gallate materials were computed using density functional theory (DFT) and Hubbard- and Hund-corrected DFT (DFT*+U+J*), performed using the pseudopotential plane-wave package implemented in Quantum ESPRESSO.^7–9^ High plane-wave cutoff energies (≥1100 eV) were used for all materials to ensure that the total energy and total interatomic forces converged to 10 meV/Å and 0.10 meV/Å, respectively. The ferrites and the gallates were sampled over 5 × 5 × 5 and 4 × 4 × 4 K-point grids, respectively. Both the Perdew, Burke, and Ernzerhof exchange-correlation functional revised for solids (PBEsol)^10,11^ and the Optimized Norm-Conserving Vanderbilt (ONCV) method^12,13^ for pseudopotentials were implemented throughout this work. Using the Broyden–Fletcher–Goldfarb–Shanno (BFGS) algorithm,^14–16^ iterative geometric relaxations were performed before computing the wave functions used for final non self-consistent field calculations (bands and densities of states).

#### Modeling Spinel Ferrites and Gallates

The relative spin alignments of adjacent paramagnetic transition metal ions in spinel oxides determine the overall magnetic ordering of the material. Ferrite materials in particular, especially CoFe_2_O_4_ and NiFe_2_O_4_, are known to have Neels ferrimagnetic ordering,^17,18^ meaning the unpaired spins of metal ions occupying O_h_ sites are aligned parallel to each other, while the unpaired spins of metal ions occupying *T*_d_ sites are aligned parallel to each other but antiparallel to the spins of the O_h_ ions. ZnFe_2_O_4_ has exhibited Neels ferrimagnetic ordering; however, it can also have antiferromagnetic ordering.^19^ For the purpose of this work, we modeled ZnFe_2_O_4_ as a normal spinel with antiferromagnetically coupled unpaired spins on the O_h_ Fe^3+^ ions. NiFe_2_O_4_ is known to preferentially crystallize fully inverted,^20,21^ meaning all the Ni^2+^ ions are octahedrally coordinated to oxygen, while half of the Fe^3+^ atoms are octahedrally coordinated and the other half are tetrahedrally coordinated. The electronic structure of NiFe_2_O_4_ was therefore calculated for the fully inverted configuration. In contrast, both CoFe_2_O_4_ and ZnFe_2_O_4_ were modeled using the normal configuration, in which all of the Co^2+^ and Zn^2+^ ions occupy *T*_d_ sites and all of the Fe^3+^ ions occupy the O_h_ sites. The gallate materials, which do not respond to an external magnetic field experimentally (*vide supra*), are modeled to have antiferromagnetic ordering of unpaired spins on open-shell metal ions. Hubbard (U) and Hund (J) parameters were calculated from first principles using a linear response to external perturbation method (see Supporting Information for details).

## Results and Discussion

### Structural Characterization of Metal Ferrites and Metal Gallates

The synthesized ferrite (Fe_3_O_4_, ZnFe_2_O_4_, NiFe_2_O_4_, and CoFe_2_O_4_) and ternary gallate (ZnGa_2_O_4_, NiGa_2_O_4_, and CoGa_2_O_4_) nanomaterials are phase-pure spinel oxides, as confirmed by powder XRD (Figure 1 and Supporting Information Figure S1). We note that although the most intense observed diffraction peaks in the γ-Ga_2_O_3_ pattern match the standard spinel structure, this diffraction pattern contains a couple of additional minor peaks that are likely a consequence of the structural disorder inherent in this cation-deficient spinel, where the relative occupancies of the tetrahedral and octahedral sites can vary within different nanocrystalline samples.^22,23^ Table 1 presents the lattice parameters (see Table S2 in the Supporting Information for calculations), particle size, and stoichiometry of spinel ferrite and gallate nanocrystals. The lattice parameters of the ternary ferrites are slightly larger than those of their gallate counterparts, reflecting differences in cation size and bonding character. Within each spinel series, a gradual contraction of the lattice parameter from Zn to Co to Ni is observed, which likely arises from a combination of differences in ionic radius and cation distribution across tetrahedral and octahedral sites within these materials (see Figure S2 in the Supporting Information for a comparison of experimental and standard lattice parameters).^3,6,24–27^ TEM imaging of these nanoparticles demonstrates that, with the exception of the binary materials Fe_3_O_4_ and γ-Ga_2_O_3_, all of the other spinel oxide nanocrystals are quasi-spherical in shape. In contrast, the Fe_3_O_4_ nanocrystals exhibit a mixture of shapes resembling triangular and rectangular prisms, with the longest visible dimension ranging from ∼20–30 nm, whereas the γ-Ga_2_O_3_ nanocrystals appear as stacks of plate-like particles with lengths ranging from ∼20–50 nm. The ternary materials all have average diameters of 6–8 nm, except for ZnFe_2_O_4_, which has an average diameter of ∼15 nm. The ternary materials are all somewhat enriched in Fe or Ga, with ratios of Fe or Ga to M ranging from 2.2 to 3.0. The standard deviation in the measured ratios across four measurements in ZnFe_2_O_4_ is ± 0.9, which is comparatively much larger than that of the other metal oxides, indicating a larger heterogeneity in metal incorporation. The colloidal solutions of all the ferrite materials are similar in color, but the color of the gallate solutions depends on the identity of the M cation. The CoGa_2_O_4_ and NiGa_2_O_4_ colloidal dispersions are distinctly blue and mint-green in color, respectively, whereas ZnGa_2_O_4_ and γ-Ga_2_O_3_ are colorless (insets in Figure 1). These data clearly indicate that upon substituting Fe for Ga in spinel oxides, the optical properties change, despite the fact that the crystal structures remain the same.

**Figure 1:**
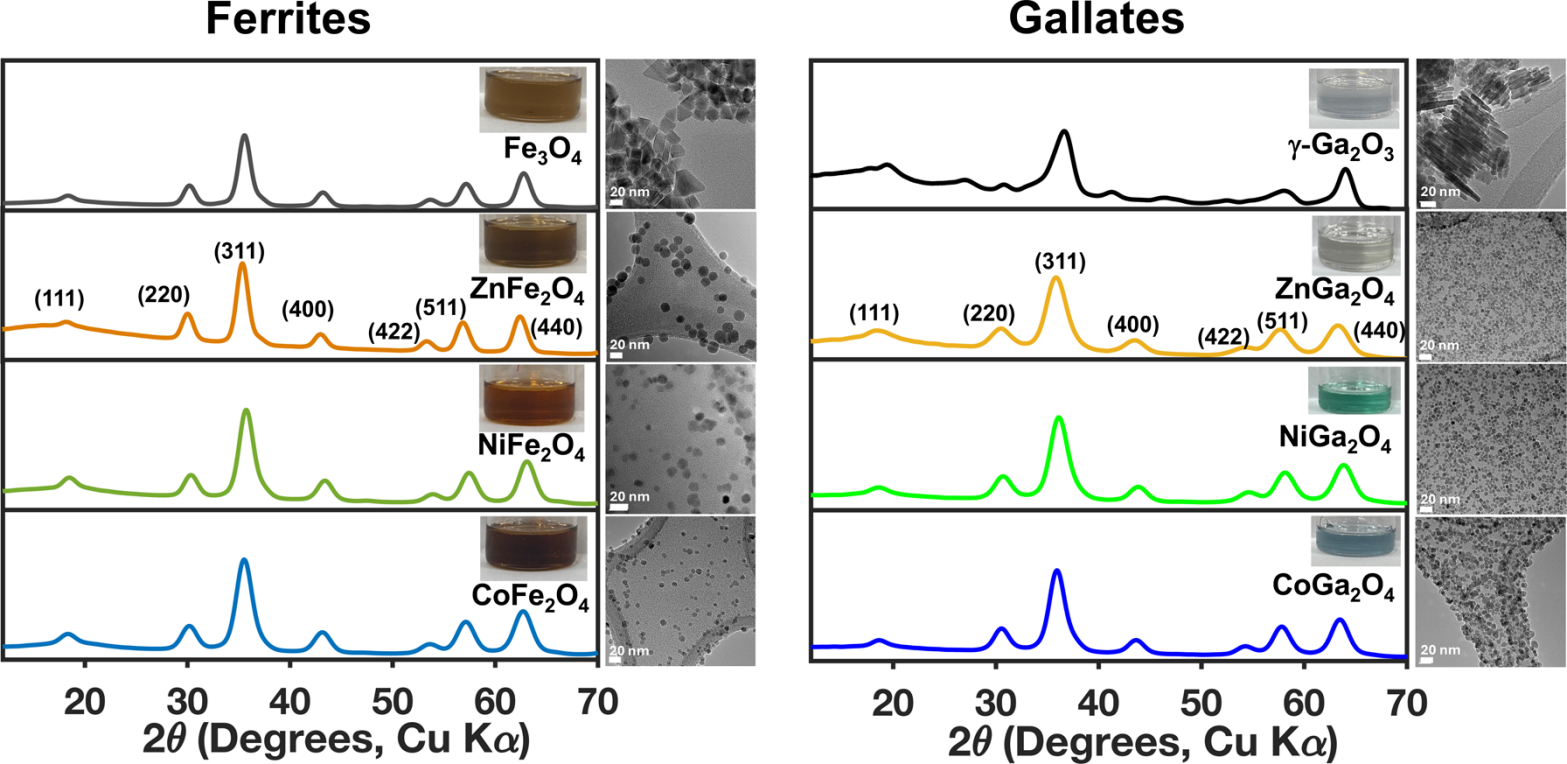
Powder XRD patterns and TEM images of as-synthesized spinel ferrite and gallate nanocrystals. The scale bars on the TEM images correspond to 20 nm. The insets contain photographs of the corresponding colloidal dispersions in tetrachloroethylene.

**Table 1:** Lattice Parameter, Particle Size, and Metal Ratios (Fe/M or Ga/M) for the Ferrite and Gallate Nanocrystals

Spinel Oxide	Lattice Parameter (Å)	Particle Size (nm)	Metal ratio
*Ferrites*			Fe:M
Fe_3_O_4_	8.40 ± 0.07	24 ± 4	-
ZnFe_2_O_4_	8.43 ± 0.02	15 ± 3	3.0 ± 0.9
NiFe_2_O_4_	8.333 ± 0.001	8.7 ± 1.7	2.3 ± 0.2
CoFe_2_O_4_	8.379 ± 0.009	7.6 ± 1.7	2.71 ± 0.09
*Gallates*			Ga:M
γ-Ga_2_O_3_	8.21 ± 0.05	34 ± 14	-
ZnGa_2_O_4_	8.32 ± 0.03	5.8 ± 0.9	2.21 ± 0.02
NiGa_2_O_4_	8.25 ± 0.01	6.2 ± 1.4	2.55 ± 0.06
CoGa_2_O_4_	8.28 ± 0.03	8.0 ± 1.6	2.4 ± 0.3

### Optical Properties and Electronic Structure of ZnGa_2_O_4_ and ZnFe_2_O_4_

Figure 2 plots the quantitative extinction spectra obtained for colloidal dispersions of ZnGa_2_O_4_, ZnFe_2_O_4_, and their binary spinel oxide counterparts, γ-Ga_2_O_3_ and Fe_3_O_4_. We note that the measured extinction spectra of γ-Ga_2_O_3_ exhibited significant intensity in the visible region, despite the fact that these dispersions were colorless (see Figure S4 in the Supporting Information). Dynamic light scattering (DLS) measurements indicate a hydrodynamic diameter of 85 nm for γ-Ga_2_O_3_ (see Figure S3 in the Supporting Information); we therefore attribute the measured extinction in the visible region to scattering. Figure 2A plots the per Ga molar extinction spectra of γ-Ga_2_O_3_ (corrected for a scattering background as detailed in the Supporting Information) and ZnGa_2_O_4_. These spectra are similar in shape, with absorption onsets around 3 eV and relatively weak intensity (ε_Ga_ < 200 M^–1^cm^–1^ over the entire range we measured). Figure 2B overlays the Fe molar extinction spectra of Fe_3_O_4_ and ZnFe_2_O_4_. Both spectra contain a low-energy transition at ∼1 eV, with the onset of continuum absorption at ∼1.6 eV. This low-energy transition in Fe_3_O_4_ is assigned to an intervalent charge transfer (IVCT) transition between Fe^2+^ and Fe^3+^ ions in octahedral sites.^28^ The intensity of the 1 eV feature depends on the concentration of iron cations in octahedrally coordinated sites.^28,29^ The iron enrichment in ZnFe_2_O_4_ (Table 1) may account for the observed low-energy transition, as it is likely that at least some of the excess iron cations have a 2+ oxidation state to maintain overall charge balance. It is also possible that some degree of cation inversion, in which iron occupies tetrahedral sites, could contribute to this absorption feature. We cannot definitively attribute the low-energy metal–metal charge transition to either iron enrichment or cation inversion, because these phenomena are not mutually exclusive; therefore, we attribute the observed transition at 1.0 eV in ZnFe_2_O_4_ to a combination of iron enrichment (in the form of excess Fe^2+^) and cation inversion. Notably, the extinction spectra of Fe_3_O_4_ and ZnFe_2_O_4_ are similar in shape but differ somewhat in the magnitude of ε_Fe_. DLS measurements of Fe_3_O_4_ indicate a hydrodynamic diameter of 160 nm compared to 26 nm for ZnFe_2_O_4_ (see Figure S3 in Supporting Information), indicating significant aggregation in the case of Fe_3_O_4_ that likely contributes to a substantial scattering background. Since Fe_3_O_4_ is brown and exhibits broad absorption throughout the visible and near-infrared regions, it is difficult to perform a quantitative scattering correction. We performed a dilution study to estimate the scattering background in Fe_3_O_4_, which supports our hypothesis that this scattering accounts for the majority of the observed difference in the magnitude of ε_Fe_ between Fe_3_O_4_ and ZnFe_2_O_4_ (see Figure S5 in Supporting Information). Overall, we conclude that the addition of Zn does not significantly alter the optical transitions in ZnFe_2_O_4_ compared to those in Fe_3_O_4_. To determine the importance of the B metal (Fe and Ga), Figure 2C plots the ε_Zn_ spectra of ZnFe_2_O_4_ and ZnGa_2_O_4_. Clearly, ZnFe_2_O_4_ has a much larger extinction coefficient, orders of magnitude larger than that of ZnGa_2_O_4_, indicating that the presence of Fe orbitals contributes to the high oscillator strength transitions of ZnFe_2_O_4_.

**Figure 2:**
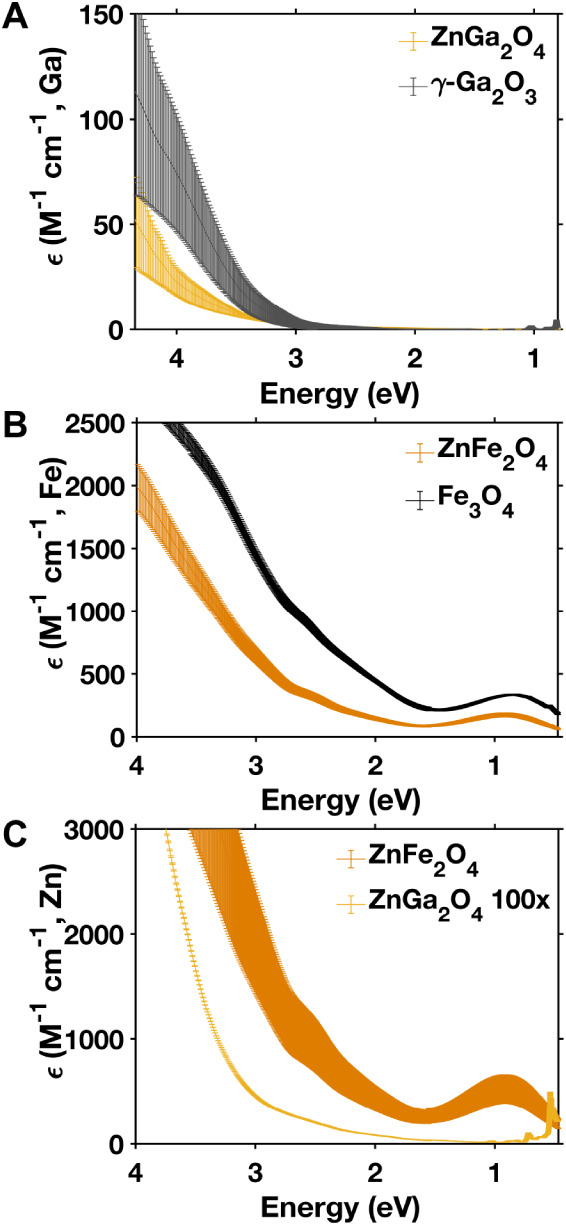
Plots of molar extinction spectra for colloidal dispersions of ZnGa_2_O_4_, γ-Ga_2_O_3_, ZnFe_2_O_4_, and Fe_3_O_4_ nanocrystals in tetrachloroethylene. Part (**A**) plots the extinction per molar concentration of Ga for ZnGa_2_O_4_ and γ-Ga_2_O_3_ , part (**B**) plots the extinction per molar concentration of Fe for ZnFe_2_O_4_ and Fe_3_O_4_ , and part (**C**) plots the extinction per molar concentration of Zn for ZnFe_2_O_4_ and ZnGa_2_O_4_. In part (**C**), the spectrum of ZnGa_2_O_4_ was multiplied by a factor of 100. The error bars originate from the standard deviation of four independent measurements obtained from two sets of syntheses (two per synthetic batch).

Figure 3 plots the electronic band structures and associated projected densities of states for ZnFe_2_O_4_ and ZnGa_2_O_4_. The projected densities of states show that bands arising from Zn do not contribute significant electronic density around the band edge, which is consistent with the experimental observation from Figure 2 that the extinction spectra of ZnFe_2_O_4_ and ZnGa_2_O_4_ are similar to those of the binary oxides Fe_3_O_4_ and γ-Ga_2_O_3_. The Zn^2+^ ions in ZnFe_2_O_4_ and ZnGa_2_O_4_ each have a full 3*d* subshell; consequently, the bands derived from these 3*d* orbitals lie deep in the valence band for both materials. In contrast, the empty Zn 4*s* orbitals sit somewhat closer to the conduction band edge; however, these 4*s* bands are much more dispersive than the 3*d* bands and thus exhibit a lower density of states. In the case of ZnFe_2_O_4_, the presence of partially filled 3*d* orbitals arising from Fe leads to weakly dispersive bands that have a large density of states over a relatively narrow energy range at the conduction band edge. In the case of ZnGa_2_O_4_, the conduction band is comprised exclusively of dispersive bands derived from Zn and Ga 4*s* orbitals, with the Ga 4*s* orbitals forming the conduction band minimum. Bands arising from oxygen 2*p* orbitals dominate the valence band edge in both ZnFe_2_O_4_ and ZnGa_2_O_4_. Thus, the large density of Fe 3*d* bands at the conduction band minimum in ZnFe_2_O_4_ gives rise to an intense ligand-to-metal charge transfer (LMCT) transition (O 2*p* → Fe 3*d*) that corresponds to the absorption onset observed experimentally at 1.6 eV (Figure 2). The comparatively smaller density of states of the more dispersive 4*s* bands that comprise the conduction band edge of ZnGa_2_O_4_ results in the much smaller extinction coefficients observed experimentally in this material. Importantly, the dispersive 4*s* orbitals contributed by Zn to the conduction band edge do little to change the optical properties of ZnFe_2_O_4_ and ZnGa_2_O_4_ relative to their binary spinel oxide counterparts (Fe_3_O_4_ and γ-Ga_2_O_3_).

**Figure 3:**
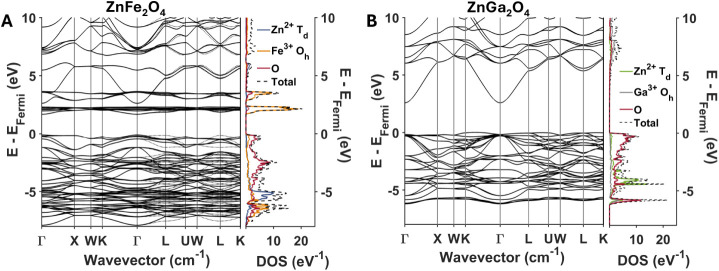
Electronic bands and projected density of states of (**A)** ZnFe_2_O_4_ and (**B)** ZnGa_2_O_4_ calculated with DFT+*U+J.*

### Optical Properties and Electronic Structure of MFe_2_O_4_ and MGa_2_O_4_ (M = Co, Ni)

Figure 4 plots the quantitative extinction spectra obtained for colloidal dispersions of CoFe_2_O_4_, NiFe_2_O_4_, CoGa_2_O_4_, and NiGa_2_O_4_. Figure 4A plots the Fe molar absorptivity spectra of Fe_3_O_4_, CoFe_2_O_4_, and NiFe_2_O_4_. Similar to ZnFe_2_O_4_, CoFe_2_O_4_, and NiFe_2_O_4_ also exhibit a low-energy transition at ∼1 eV, with the onset of the highest oscillator strength transition at ∼1.6 eV. However, the intensity of the 1-eV feature decreases with a decrease in the iron concentration.^28^ The iron content in CoFe_2_O_4_ and NiFe_2_O_4_ is less than Fe_3_O_4_, hence, the decrease in the intensity of the 1 eV feature. Furthermore, the iron enrichment in the CoFe_2_O_4_ and NiFe_2_O_4_ samples is also less than that observed for the ZnFe_2_O_4_ samples, indicating that CoFe_2_O_4_ and NiFe_2_O_4_ contain a smaller concentration of excess Fe^2+^. This smaller concentration of excess Fe^2+^ is consistent with the smaller intensity of the 1 eV feature observed in CoFe_2_O_4_ and NiFe_2_O_4_ compared to ZnFe_2_O_4_. In contrast, the per Ga molar extinction spectra of CoGa_2_O_4_ and NiGa_2_O_4_, shown in Figure 4C are distinct from each other, and both differ from the spectrum of γ-Ga_2_O_3_. Both CoGa_2_O_4_ and NiGa_2_O_4_ contain absorption features consistent with intra-atomic *d-d* transitions of cobalt and nickel, respectively: CoGa_2_O_4_ contains absorption features consistent with Co^2+^ in a tetrahedral crystal field, and NiGa_2_O_4_ contains absorption features consistent with Ni^2+^ in an octahedral crystal field.^30^ Figure 4B and D plot the per Co and per Ni extinction spectra of CoFe_2_O_4_, NiFe_2_O_4_ and CoGa_2_O_4_, NiGa_2_O_4_, respectively. These spectra further highlight how the *d-d* transitions dominate the spectra of the gallates, whereas the ferrite spectra are dominated by broad O 2*p* → 3*d* charge transfer transitions. Furthermore, the per Co and per Ni molar absorptivities of the ferrites are ∼2 orders of magnitude larger than those of the gallates, which is consistent with the assignments of these features to charge transfer and *d-d* transitions, respectively.

**Figure 4:**
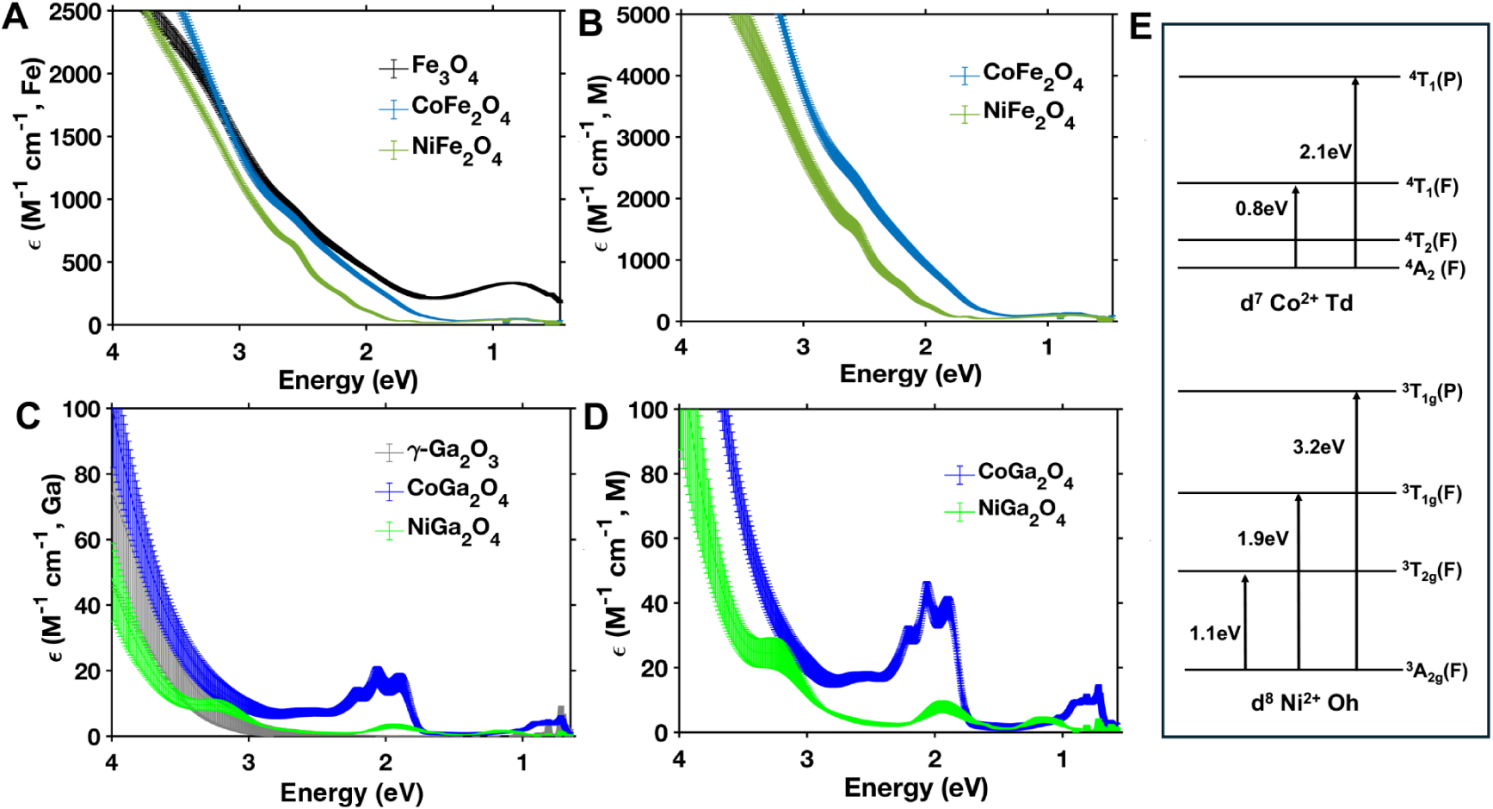
(**A–D**) Plots of molar extinction spectra for colloidal dispersions of Fe_3_O_4_, CoFe_2_O_4_, NiFe_2_O_4_, γ-Ga_2_O_3_, CoGa_2_O_4_, and NiGa_2_O_4_ nanocrystals in tetrachloroethylene. Part (**A**) plots the extinction per molar concentration of Fe for Fe_3_O_4_, CoFe_2_O_4_, and NiFe_2_O_4_, part (**B**) plots the extinction per molar concentration of the M metal (Co or Ni) for CoFe_2_O_4_ and NiFe_2_O_4_, part (**C**) plots the extinction per molar concentration of Ga for γ-Ga_2_O_3_, CoGa_2_O_4_, and NiGa_2_O_4_, and part (**D**) plots the extinction per molar concentration of the M metal (Co or Ni) for CoGa_2_O_4_ and NiGa_2_O_4._ The error bars originate from the standard deviation of four independent measurements obtained from two sets of syntheses (two per synthetic batch). (**E**) Tanabe-Sugano energy-level schemes for Co^2+^ in a tetrahedral field (*top*) and Ni^2+^ in an octahedral field (*bottom*).

Upon consulting a Tanabe–Sugano energy level diagram (Figure 4E) for a d^7^ ion (Co^2+^) in a tetrahedral crystal field, the visible absorption band around 2.1 eV in CoGa_2_O_4_ can be assigned to the ^4^A_2_ → ^4^T_1_ (^4^P) transition, and the infrared band at about 0.8 eV can be assigned to the ^4^A_2_ → ^4^T_1_ (^4^F) transition (Figure 4E). These features even exhibit higher-order splitting due to spin–orbit and vibronic coupling.^31,32^ For NiGa_2_O_4_, the spectrum is dominated by three spin-allowed broad-band transitions that can be assigned using the Tanabe–Sugano diagram for a d^8^ ion in an octahedral crystal field. These transitions are ^3^A_2g_(^3^F) → ^3^T_1g_ (^3^P) in the deep blue around 3.2 eV, ^3^A_2g_(^3^F) → ^3^T_1g_ (^3^F) in the red around 1.9 eV, and ^3^A_2g_(^3^F) → ^3^T_2g_ (^3^F) in the near-infrared around 1.1 eV.^30,33^ These transitions are also visible in the spectrum of NiFe_2_O_4_ as small perturbations on top of the more intense O 2*p* → Fe 3*d* charge-transfer transitions.

Figure 5 plots the projected densities of states calculated for CoFe_2_O_4_, NiFe_2_O_4_, CoGa_2_O_4_, and NiGa_2_O_4_ using DFT+*U* + *J*. The unpaired spin on the 3*d* metals in the ferrite materials is modeled with Neels ferrimagnetic ordering. The Neels model aligns the unpaired spins of atoms in O_h_ sites in one direction and those in *T*_d_ sites in the opposite direction.^17,18^ With this ferrimagnetic ordering, both CoFe_2_O_4_ and NiFe_2_O_4_ have nonsymmetric spin channels in their projected density of states, leading to spin-selective transitions. Like ZnFe_2_O_4_, the valence and conduction band edges of these ferrites contain contributions from O 2*p* and Fe 3*d* orbitals, respectively; however, unlike ZnFe_2_O_4_, the M metals (Co or Ni) also contribute 3*d* bands to both conduction and valence band edges. The presence of two open-shell metals in the spinel structure (Fe^3+^ and Co^2+^ or Fe^3+^ and Ni^2+^) leads to the possibility of metal-to-metal charge transfer (MMCT) transitions. In CoFe_2_O_4_, for example, a transition from *T*_d_ Co^2+^ spin down → O_h_ Fe^3+^ spin down has the same energy as the O 2*p* spin down → O_h_ Fe^3+^ spin down transition. Both of these transitions are allowed by optical selection rules and energetically match the high-oscillator strength transition with an onset at ∼1.6 eV observed in the absorption spectrum of colloidal CoFe_2_O_4_ nanoparticles. Similarly, in NiFe_2_O_4_, a transition from O_h_ Ni^2+^ spin down → *O*_h_ Fe^3+^ spin down could also contribute to the band-edge absorption.

**Figure 5:**
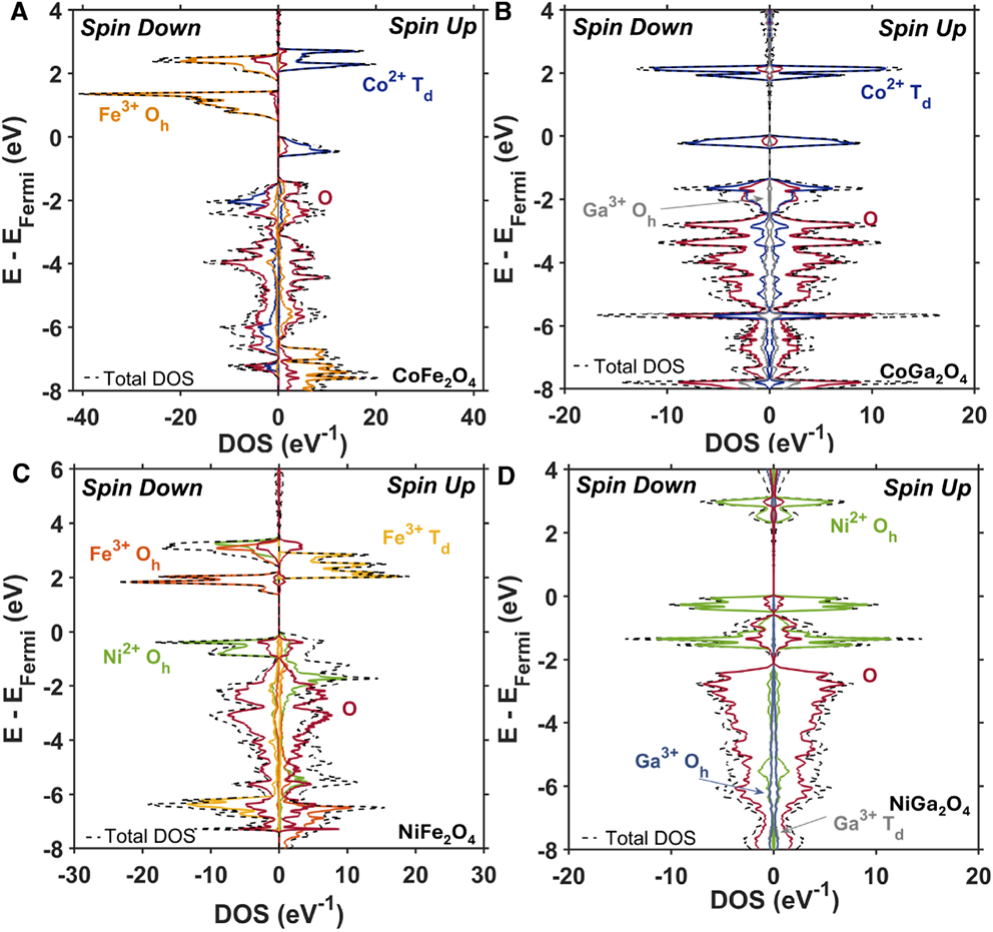
Electronic projected density of states of (**A)** normal CoFe_2_O_4_, (**B)** normal CoGa_2_O_4_, (**C)** fully inverted NiFe_2_O_4_, and (**D)** fully inverted NiGa_2_O_4_ calculated with DFT+*U* + *J*.

Comparison of the projected density of states of CoGa_2_O_4_ to CoFe_2_O_4_ (Figure 5A–B) demonstrates that, without the presence of the high electronic density arising from Fe 3*d* orbitals, the band-edge of CoGa_2_O_4_ is dominated by Co electronic character. As observed in ZnGa_2_O_4_, Ga contributes to dispersive conduction bands. With the addition of an open-shell metal to the gallate structure, the weakly dispersive bands arising from Co orbitals make up the majority of the band-edge character (see Supporting Information for complete band structures). The electronic density of Fe in the conduction band (spin down) of CoFe_2_O_4_ is almost 2-fold higher than that of Co in CoGa_2_O_4_. However, the densities of Co states in the conduction bands of CoFe_2_O_4_ and CoGa_2_O_4_ are similar, indicating that Co is contributing similar electronic character in both materials. Additionally, the calculated interatomic energy gap from *T*_d_ Co^2+^ in the valence band to the conduction band is the same in both CoGa_2_O_4_ and CoFe_2_O_4_ (spin up); however, because Fe electronic character dominates the CoFe_2_O_4_ band-edge, these *d-d* transitions are less prominent in the experimental absorption spectrum. As shown experimentally, the per M (Co) molar absorptivity spectra of CoFe_2_O_4_ is 2 orders of magnitude larger than that of CoGa_2_O_4_ (Figure 4B, D), suggesting that, despite having similar calculated densities of Co states at the band-edge, it is the O 2*p* → O_h_ Fe^3+^ transitions in CoFe_2_O_4_ that contribute the most to the intensity of the absorption spectrum.

Both NiFe_2_O_4_ and NiGa_2_O_4_ were modeled as fully inverted to account for the weakly allowed O_h_ Ni^2+^ *d-d* transitions observed in experimental spectra of both colloidal nanocrystal samples (Figure 4). When Ni is octahedrally coordinated, it should be noted that the intra-atomic *d-d* transitions are Laporte-forbidden unless there are vibrational distortions making them weakly allowed, which have been observed in other nickel oxide materials.^34,35^ With fully inverted crystal structures, half of the B metal ions (Fe or Ga) are octahedrally coordinated, and the other half are tetrahedrally coordinated, while all the M metal ions (Ni) are octahedrally coordinated. Because NiFe_2_O_4_ is modeled to have Neel’s ferrimagnetic ordering, there are two distinct spin channels that dictate optical transitions. Conversely, because of the antiferromagnetic ordering of unpaired spin in NiGa_2_O_4_ the overall spin-resolved electronic density is symmetric. Although cation inversion in NiFe_2_O_4_ complicates the electronic transitions, the same trend can be observed as in CoFe_2_O_4_: there is a high density of Fe states at the conduction band edge. However, the valence band edge of NiFe_2_O_4_ is dominated by Ni character in the spin-down channel, with nearly equal contributions of Ni and oxygen character in the spin-up channel. In NiGa_2_O_4_, Ni character dominates both the conduction and valence band edges with Ga contributing at higher energies in the conduction band, as observed in CoGa_2_O_4_. We therefore conclude that ternary oxides with only one open-shell transition metal (CoGa_2_O_4_ and NiGa_2_O_4_) have pronounced *d*-*d* transitions compared to ternary oxides with two open-shell transition metals (CoFe_2_O_4_ and NiFe_2_O_4_) because the strong LMCT transition from O to Fe is not present in the gallates. We predict this trend is not specific to the gallate/ferrite families of ternary spinel materials but rather showcases how the spinel crystal structure provides the ability to tune optical transitions based on composition.^36,37^

### Implications for Photocatalysis

The insights revealed by this quantitative analysis of the optical properties of colloidal ternary spinel ferrite and gallate nanocrystals have broad implications for the design of nanocrystal photocatalysts. They indicate that, for metal ferrites of formula MFe_2_O_4_, the majority of the visible absorption arises from transitions into Fe 3*d* bands, regardless of the identity of the M metal. These transitions largely comprise ligand-to-metal charge transfer (LMCT) type transitions from O 2*p* to Fe 3*d*, and they have relatively large extinction coefficients. The presence of other metal 3*d* bands at the valence band edge (e.g., as in CoFe_2_O_4_ and NiFe_2_O_4_) can lead to additional contributions from metal-to-metal charge transfer (MMCT) type transitions. However, the dominance of Fe 3*d* bands in the conduction band for these ferrites suggests that the identity of the M metal may have a minimal impact on photocatalytic performance in processes that rely on photoinduced electron transfer, since the photogenerated electron initially occupies an Fe 3*d* band in every case. In contrast, the identity of the M metal can impact the orbital character of the valence band edge and thus may have a larger impact on photocatalytic performance in processes that rely on photoinduced hole transfer. Our group recently reported a comparative study of the ability of a series of ternary spinel ferrite nanocrystals to photocatalyze degradation of a model organic dye molecule via the photo-Fenton mechanism, which involves photoinduced electron transfer from the ferrite nanocrystal to hydrogen peroxide.^38^ We found significant differences in the photocatalytic activity across the different ferrite materials. Taken together with the results reported here revealing the universality of Fe 3*d* bands at the conduction band edge in metal ferrites, these two studies suggest that additional factors other than absorption properties, such as surface chemistry or cation distribution, may play a decisive role in influencing the photocatalytic activity of spinel ferrite nanocrystals. With respect to the cation distribution, we note that Figure 5C indicates that the conduction band edge in inverted NiFe_2_O_4_ has both Fe^3+^ *T*_d_ and Fe^3+^ O_h_ 3*d* bands. Whether the presence of these two different bands significantly impacts the photophysics and photocatalytic performance of inverted spinel ferrites is an open question we are currently pursuing.

Unlike the spinel ferrites, spinel gallate nanocrystals exhibit very different absorption spectra with different M^2+^ metals. Any absorption features observed in the visible region for these materials arise from localized intraatomic *d*-*d* transitions within the M^2+^ cation, as observed for CoGa_2_O_4_ and NiGa_2_O_4_. In the absence of such transitions, the materials are colorless, with no absorption in the visible region, as observed for γ-Ga_2_O_3_ and ZnGa_2_O_4_. Compared to the charge transfer transitions present in the spinel ferrites, these transitions have much smaller absorption cross-sections, with per Ga extinction coefficients that are more than a factor of 100 smaller than the per Fe extinction coefficients measured for the ferrites. Furthermore, the *d*-*d* transitions are less conducive to charge separation than the charge transfer transitions, which is also less desirable for photocatalysis. However, despite these disadvantages, CoGa_2_O_4_ nanocrystals have been reported to engage in photocatalytic activity upon visible excitation, demonstrating that these *d*-*d* transitions are capable of driving photochemistry.^39^

## Conclusions

This work reveals that changing the identity of the M metal in colloidal MFe_2_O_4_ or MGa_2_O_4_ nanocrystals has varying impacts on their optical properties. Changing the identity of the M metal in MFe_2_O_4_ has minimal impact on the onset of band-edge absorption because these transitions primarily arise from strong O 2*p* → Fe 3*d* charge-transfer processes, regardless of the identity of M. In contrast, replacing Fe with Ga to form ternary metal oxides of formula MGa_2_O_4_ reveals comparatively weaker sub-band gap *d-d* transitions with energies that depend on the identity and geometry of M^2+^. Since Ga^3+^ does not have any partially filled 3*d* orbitals, and the O 2*p* → Ga 4*s* charge-transfer transition occurs at very high energy (deep UV), the only transitions that occur in the visible region arise from *d-d* transitions corresponding to M^2+^, such as Co^2+^ in CoGa_2_O_4_ and Ni^2+^ in NiGa_2_O_4_. In CoGa_2_O_4_, these transitions correspond to crystal-field transitions of *T*_d_ Co^2+^ and in NiGa_2_O_4_, these transitions correspond to crystal-field transitions of O_h_ Ni^2+^. No visible absorptions were observed in ZnGa_2_O_4_ because Zn^2+^ has a d^10^ configuration that precludes *d*-*d* transitions. In ferrites, all weak *d*-*d* transitions arising from Fe^3+^ are spin-forbidden, and the high oscillator strength of the O 2*p* → Fe^3+^ 3*d* charge-transfer absorption masks *d-d* transitions arising from the M^2+^ ions. Although this work clearly demonstrates the impacts of changing composition on the optical properties of ternary spinel oxide nanocrystals, the impacts of different distributions of cations among octahedral and tetrahedral sites have yet to be investigated. Understanding these relationships and their impact on the photophysics of ternary spinel oxide nanocrystals is crucial to the development of these materials as photoactive materials in catalysis and sensing applications.

## Supplementary Material



## Notes

Additional experimental details describing lattice parameter determination; sample preparation procedures for AA and ICP measurements; dynamic light scattering and scattering background correction in Fe_3_O_4_ and γ-Ga_2_O_3_; calculation of Hubbard *U* and Hund *J* parameters using a linear response perturbation method; and plots of complete band structures for MFe_2_O_4_ and MGa_2_O_4_ (M = Co, Ni, Zn) (PDF)
