# Supplementary material for: Impact of Composition on the Optical Properties of Colloidal Ternary Spinel Oxide Nanocrystals: Spinel Ferrites versus Spinel Gallates
Source: Chem Mater. 2026 Apr 30;38(9):4561–9. doi: 10.1021/acs.chemmater.5c03447 (PMC13173498; doi:10.1021/acs.chemmater.5c03447)
Supplement: Supplementary file 1 [file cm5c03447_si_001.pdf]

Supplementary Information

Impact of Composition on the Optical Properties of Colloidal Ternary Spinel Oxide Nanocrystals: Spinel Ferrites versus Spinel Gallates

Revathy Rajan, Erica P. Craddock, Kathryn E. Knowles*

Department of Chemistry, University of Rochester, Rochester, New York 14627, United States

*corresponding author. E-mail: kknowles@ur.rochester.edu

Sample Preparation for Atomic Absorption (AA) and ICP-MS Measurements.

Concentrated stock solutions in tetrachloroethylene (TCE) was made of each metal ferrite and metal gallate nanocrystal sample according to the mass-to-volume concentrations listed in Table S1. To prevent signal saturation in the absorption measurement and obtain accurate absorbance values, many of the stock solutions were diluted with TCE prior to collecting absorption spectra. The relevant dilution factors are listed in Table S1. To prepare samples for AA or ICP analysis, small volumes of each concentrated stock solution were dried under N_2 to remove the solvent, and the remaining solids were digested in 0.5 mL of concentrated nitric acid or aqua regia before dilution to a total volume of 15 mL with Nanopure water. Table S1 lists the volumes of the stock solution used for each sample. These samples were characterized by either AA for ferrites or ICP-MS for gallates to obtain the metal concentrations. These concentrations were determined using calibration curves measured for solutions of known metal ion concentration, which were prepared by diluting commercially available standard solutions (High-Purity Standards). Using the dilution factors reported in Table S1, we extrapolated the concentration of the metals present in the solutions characterized by UV-Vis absorption. These are the concentrations reported in Table S1 and used in the Beer-Lambert equation ($A = \varepsilon c l$) to compute extinction coefficients.

Table S1. Parameters used to prepare samples for AA and ICP measurements

Sample	Stock Concentration (mg/ml)	Dilution factor for UV-Vis	Volume used for AA/ICP Sample (μL)	Fe or Ga concentration (M)	Metal concentration (M)
Fe_3O_4	1	10	50	5.27×10^{-4}	-
			75	5.46×10^{-4}	-
			50	2.96×10^{-4}	-
			75	3.00×10^{-4}	-
$CoFe_2O_4$	1	10	50	5.28×10^{-4}	1.94×10^{-4}
			75	5.24×10^{-4}	2.03×10^{-4}
			50	5.32×10^{-4}	1.90×10^{-4}
			75	5.54×10^{-4}	2.02×10^{-4}
$NiFe_2O_4$	5	50	25	1.09×10^{-3}	3.36×10^{-4}
			30	1.12×10^{-3}	3.12×10^{-4}
			25	7.89×10^{-4}	4.99×10^{-4}
			30	7.89×10^{-4}	4.47×10^{-4}
$ZnFe_2O_4$	1	10	25	7.60×10^{-4}	3.81×10^{-4}
			50	8.56×10^{-4}	2.17×10^{-4}
			25	8.01×10^{-4}	2.60×10^{-4}
			50	9.10×10^{-4}	2.93×10^{-4}
γ-Ga_2O_3	5	5	25	1.13×10^{-3}	-
			30	1.18×10^{-3}	-
			25	2.04×10^{-3}	-
			30	2.14×10^{-3}	-
$CoGa_2O_4$	5	1	50	3.01×10^{-2}	1.36×10^{-2}
			100	2.94×10^{-2}	1.34×10^{-2}
			25	2.77×10^{-2}	1.18×10^{-2}
			30	3.97×10^{-2}	1.43×10^{-2}
$NiGa_2O_4$	10	1	25	6.48×10^{-2}	2.62×10^{-2}
			30	6.65×10^{-2}	2.62×10^{-2}
			25	3.95×10^{-2}	1.52×10^{-2}
			30	3.83×10^{-2}	1.48×10^{-2}
$ZnGa_2O_4$	3	1	50	1.13×10^{-2}	5.07×10^{-3}
			100	9.41×10^{-3}	4.28×10^{-3}
			50	1.53×10^{-2}	6.91×10^{-3}
			100	1.54×10^{-2}	6.92×10^{-3}

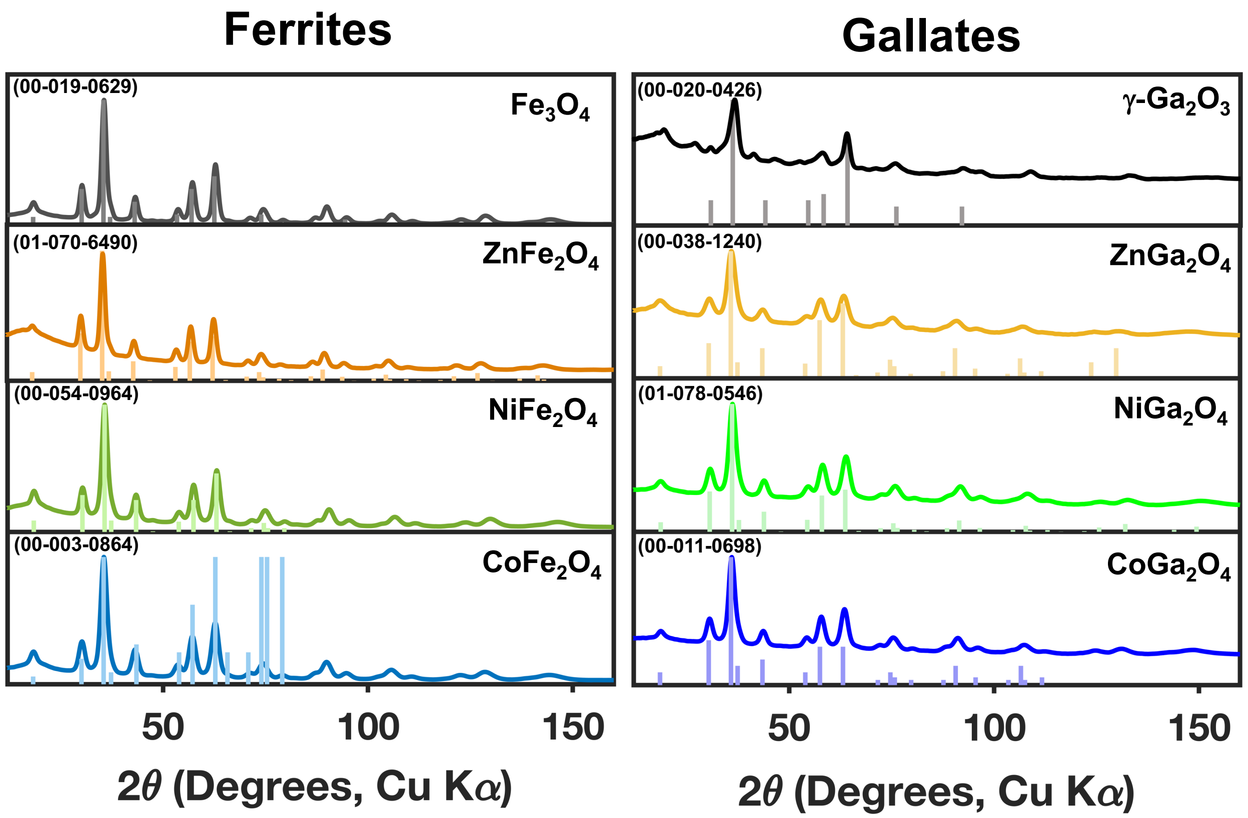

Figure S1. Powder XRD patterns of as-synthesized spinel ferrite and gallate nanocrystals. The solid vertical lines in the XRD patterns represent standard patterns for these materials extracted from the JCPDS database (JCPDS entry numbers are inset into the upper left-hand corners of each diffraction pattern plot.

Lattice Parameter Calculation

The lattice parameters (a) of all the ferrite and gallate nanocrystals were determined from the peak positions in the powder X-ray diffraction patterns obtained after conversion to Cu Kα radiation (λ = 1.54158 Å) using equations S1 and S2.

$$d_{hkl} = \frac{\lambda}{2sin\theta} \quad \text{(S1)}$$

$$a = d_{hkl}\sqrt{h^2 + k^2 + l^2} \quad \text{(S2)}$$

In these equations, λ is the wavelength of the X-ray source, d_{hkl} is the d-spacing between the crystal planes (hkl), θ is the Bragg angle, and *hkl* are the Miller indices associated with each diffraction peak. We calculated values of lattice parameters from seven peaks in the XRD pattern for all

nanocrystals (exception: γ-Ga_2O_3, 5 peaks) and report their average in the main text. Table S2 contains the complete set of data used in these calculations for all the nanocrystals.

Table S2. Powder X-ray diffraction data used to determine the Lattice Parameter of the Ferrite and the Gallate nanocrystals

Fe_3O_4

λ (Å)	Miller indices			Bragg's angle (°)		d-spacing (Å)	$\sqrt{(h^2+k^2+l^2)}$	Lattice constant (Å)	Average a (Å)
	h	k	l	2θ	θ				
1.54158	1	1	1	18.34	9.17	4.8361	1.7320	8.376	
1.54158	2	2	0	30.18	15.09	2.9604	2.8284	8.373	
1.54158	3	1	1	35.42	17.71	2.5336	3.3166	8.403	8.403
1.54158	4	0	0	42.19	21.09	2.1414	4	8.565	
1.54158	4	2	2	53.68	26.84	1.7071	4.8989	8.363	
1.54158	5	1	1	57.15	28.57	1.6112	5.1961	8.372	
1.54158	4	4	0	62.80	31.40	1.4794	5.6568	8.368	

$CoFe_2O_4$

λ (Å)	Miller indices			Bragg's angle (°)		d-spacing (Å)	$\sqrt{(h^2+k^2+l^2)}$	Lattice constant (Å)	Average a (Å)
	h	k	l	2θ	θ				
1.54158	1	1	1	18.34	9.17	4.8361	1.7320	8.376	
1.54158	2	2	0	30.18	15.09	2.9604	2.8284	8.373	
1.54158	3	1	1	35.45	17.72	2.5317	3.3166	8.396	8.379
1.54158	4	0	0	43.14	21.57	2.0962	4	8.385	
1.54158	4	2	2	53.63	26.81	1.7085	4.8989	8.370	
1.54158	5	1	1	57.13	28.56	1.6118	5.1961	8.375	
1.54158	4	4	0	62.70	31.35	1.4815	5.6568	8.380	

$NiFe_2O_4$

λ (Å)	Miller indices			Bragg's angle (°)		d-spacing (Å)	$\sqrt{(h^2+k^2+l^2)}$	Lattice constant (Å)	Average a (Å)
	h	k	l	2θ	θ				
1.54158	1	1	1	18.45	9.22	4.8077	1.7320	8.327	
1.54158	2	2	0	30.33	15.16	2.9455	2.8284	8.331	
1.54158	3	1	1	35.70	17.85	2.5145	3.3166	8.339	8.333
1.54158	4	0	0	43.37	21.68	2.0856	4	8.342	
1.54158	4	2	2	53.94	26.97	1.6994	4.8989	8.325	
1.54158	5	1	1	57.44	28.72	1.6038	5.1961	8.333	
1.54158	4	4	0	63.09	31.54	1.4731	5.6568	8.333	

$ZnFe_2O_4$

λ (Å)	Miller indices			Bragg's angle (°)		d-spacing (Å)	$\sqrt{(h^2+k^2+l^2)}$	Lattice constant (Å)	Average a (Å)
	h	k	l	2θ	θ				
1.54158	1	1	1	18.16	9.08	4.8841	1.7320	8.459	
1.54158	2	2	0	30.02	15.01	2.9754	2.8284	8.415	
1.54158	3	1	1	35.23	17.61	2.5467	3.3166	8.446	8.426
1.54158	4	0	0	42.94	21.47	2.1058	4	8.423	
1.54158	4	2	2	53.32	26.66	1.7177	4.8989	8.415	
1.54158	3	3	3	56.86	28.43	1.6187	5.1961	8.411	
1.54158	4	4	0	62.40	31.20	1.4878	5.6568	8.416	

γ-Ga_2O_3

λ (Å)	Miller indices			Bragg's angle (°)		d-spacing (Å)	$\sqrt{(h^2+k^2+l^2)}$	Lattice constant (Å)	Average a (Å)
	h	k	l	2θ	θ				
1.54158	2	2	0	30.80	15.40	2.9021	2.8284	8.208	
1.54158	3	1	1	36.70	18.35	2.4482	3.3166	8.119	
1.54158	5	1	1	57.99	28.99	1.5900	5.1961	8.261	8.207
1.54158	4	4	0	64.05	32.02	1.4534	5.6568	8.221	
1.54158	5	3	3	75.85	37.92	1.2540	6.5574	8.223	

$CoGa_2O_4$

λ (Å)	Miller indices			Bragg's angle (°)		d-spacing (Å)	$\sqrt{(h^2+k^2+l^2)}$	Lattice constant (Å)	Average a (Å)
	h	k	l	2θ	θ				
1.54158	1	1	1	18.66	9.33	4.7544	1.7320	8.234	
1.54158	2	2	0	30.54	15.27	2.9263	2.8284	8.276	
1.54158	3	1	1	35.74	17.87	2.5112	3.3166	8.328	8.283
1.54158	4	0	0	43.62	21.81	2.0746	4	8.298	
1.54158	4	2	2	53.30	27.15	1.6889	4.8989	8.274	
1.54158	5	1	1	57.83	28.91	1.5941	5.1961	8.283	
1.54158	4	4	0	63.49	31.74	1.4648	5.6568	8.286	

$NiGa_2O_4$

λ (Å)	Miller indices			Bragg's angle (°)		d-spacing (Å)	$\sqrt{(h^2+k^2+l^2)}$	Lattice constant (Å)	Average a (Å)
	h	k	l	2θ	θ				
1.54158	1	1	1	18.60	9.30	4.7682	1.7320	8.258	
1.54158	2	2	0	30.72	15.36	2.9099	2.8284	8.230	
1.54158	3	1	1	36.02	18.01	2.4924	3.3166	8.266	8.249
1.54158	4	0	0	43.81	21.90	2.0658	4	8.263	
1.54158	4	2	2	54.54	27.27	1.6822	4.8989	8.241	
1.54158	5	1	1	58.12	29.06	1.5867	5.1961	8.244	
1.54158	4	4	0	63.86	31.93	1.4572	5.6568	8.243	

$ZnGa_2O_4$

λ (Å)	Miller indices			Bragg's angle (°)		d-spacing (Å)	$\sqrt{(h^2+k^2+l^2)}$	Lattice constant (Å)	Average a (Å)
	h	k	l	2θ	θ				
1.54158	1	1	1	18.36	9.18	4.8304	1.7320	8.366	
1.54158	2	2	0	30.45	15.22	2.9349	2.8284	8.301	
1.54158	3	1	1	35.69	17.84	2.514	3.3166	8.340	8.318
1.54158	4	0	0	43.53	21.76	2.0782	4	8.313	
1.54158	4	2	2	54.22	27.11	1.6911	4.8989	8.284	
1.54158	5	1	1	57.64	28.82	1.5989	5.1961	8.308	
1.54158	4	4	0	63.24	31.62	1.4700	5.6568	8.315	

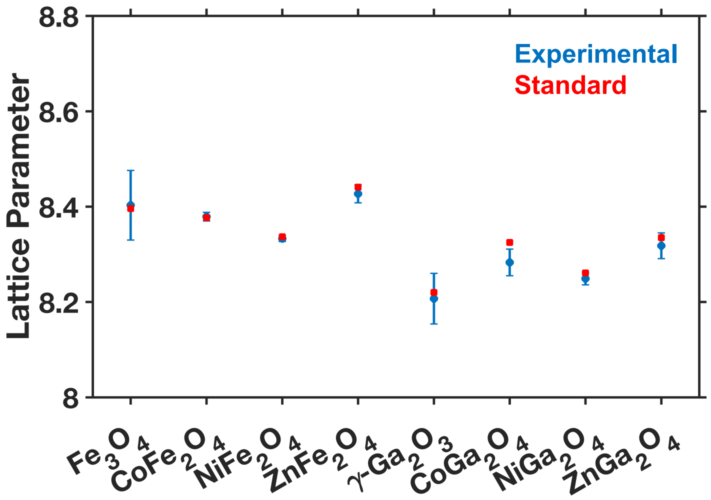

Figure S2. Experimental and standard lattice parameters (from same JCPDS files used in Figure S1) of the different ferrites and gallates.

Dynamic Light Scattering

Dynamic light scattering (DLS) measurements were carried out using a DynaPro Nanostar II (Wyatt Technology) at room temperature and the data processing was performed using the "Dynamics^{TM} 8" software. The concentration of the different ferrites and gallates was the same as that used for collecting the absorption spectra (Table S1). All measurements were performed in triplicate in a glass cuvette, and the reported hydrodynamic diameters represent the average value with standard deviation obtained from replicate measurements.

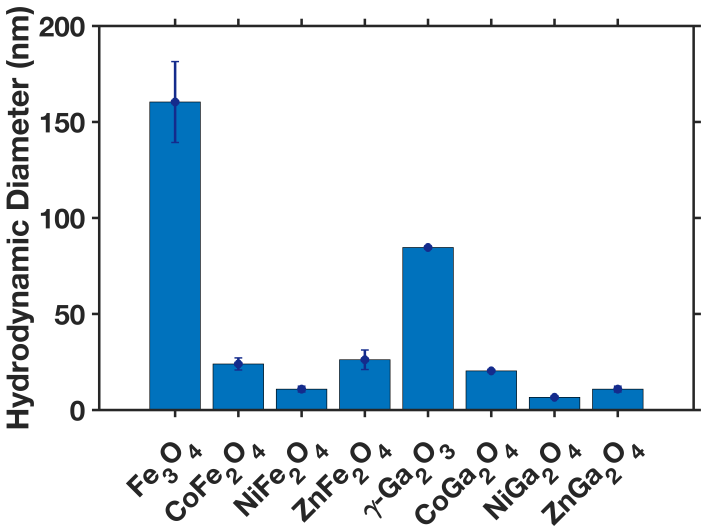

Figure S3. Hydrodynamic diameter of the different ferrites and gallates in TCE obtained from Dynamic Light Scattering. The error bars originate from the standard deviation of three measurements.

Scattering background correction in Fe_3O_4 and γ-Ga_2O_3

The hydrodynamic diameters of the binary oxides Fe_3O_4 and γ-Ga_2O_3 obtained from DLS are comparatively much larger than their ternary counterparts, suggesting the likelihood of significant contributions of scattering to the extinction spectra of the colloidal solutions of these nanocrystals. Furthermore, since γ-Ga_2O_3 is completely colorless, absorption in the visible region (400-800 nm) should be negligible. Therefore, we attribute the extinction observed in this region to scattering and account for it by performing the following scattering correction procedure. The measured extinction was modeled as the sum of absorption and a wavelength-dependent scattering background (equation S3). We used a power law model (equation S4) to describe the scattering background where k is the scattering amplitude, n is the scattering exponent describing the wavelength dependence, and b is a wavelength-independent baseline offset.[1,2]

$$\epsilon_{measured}(\lambda) = \epsilon_{abs}(\lambda) + \epsilon_{scatt}(\lambda) \quad \text{(S3)}$$

$$\epsilon_{scatt}(\lambda) = k\lambda^{-n} + b \quad \text{(S4)}$$

In the region from 600 nm to 1000 nm, where absorption is expected to be minimal, we assume that the measured extinction is entirely due to scattering ($\epsilon_{measured}(\lambda) \approx \epsilon_{scatt}(\lambda)$). We used the Solver package in Microsoft Excel to perform a nonlinear least-squares minimization to fit this

region of the extinction spectrum to equation S4. Constraints of $k \geq 0$ and $0 \leq n \leq 4$ were applied to the least-squares minimization. The fitted scattering background was then subtracted from the measured spectrum to obtain the scatter-corrected molar extinction spectrum of γ-Ga_2O_3. This fitting procedure was performed for four measured extinction spectra of γ-Ga_2O_3 as shown in Figure S4, and the average corrected epsilon with the standard deviation is plotted in Figures 2 and 4 in the main text. Table S3 contains the fit parameters obtained using nonlinear least-squares minimization.

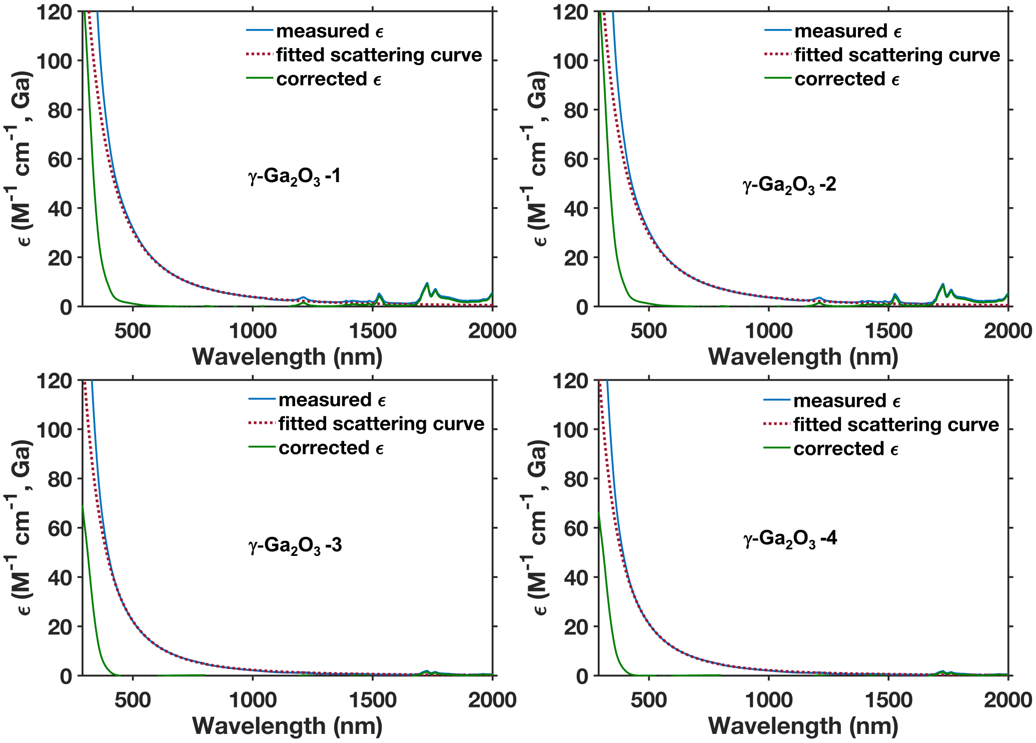

Figure S4. Molar extinction spectra of four samples of γ-Ga_2O_3 nanocrystals (1-4). The measured spectra (blue) were fitted in the wavelength region 600 nm to 1000 nm, where absorption is negligible, to a power-law scattering model described by equation S4. The fitted scattering background (dotted red) and the corrected spectra obtained after background subtraction (green) are shown.

Table S3. Parameters obtained using nonlinear least-squares minimization to fit the scattering backgrounds of the γ-Ga_2O_3 extinction spectra.

Sample	*k*	*n*	*b*
γ-Ga_2O_3-1	3.58×10^9	2.99	0
γ-Ga_2O_3-2	3.21×10^9	2.98	0
γ-Ga_2O_3-3	1.59×10^{10}	3.28	0
γ-Ga_2O_3-4	1.48×10^{10}	3.28	0

The DLS data shown in Figure S3 indicate that the Fe_3O_4 samples experience significant aggregation in 0.1 mg/mL colloidal dispersions and the resulting scattering may account for the observed differences in the magnitudes of the per-Fe extinction coefficients observed for Fe_3O_4 and the ternary ferrites. However, since Fe_3O_4 exhibits absorption throughout the visible and near-infrared regions, it is difficult to identify a wavelength range where absorption is negligible, and the resulting fit parameters are therefore less uniquely constrained. Instead, we estimated the scattering contribution to the Fe_3O_4 extinction spectrum using a dilution experiment. Absorbance spectra for a sample of Fe_3O_4 nanocrystals were collected at two different concentrations (0.1 mg/ml, and a 4x dilution of this dispersion). The diluted spectrum was compared to the concentrated spectrum divided by the dilution factor of 4. The difference between the diluted spectrum and the concentrated spectrum scaled down by a linear dilution factor of 4 fit to equation S3 using the same Excel Solver fitting procedure applied to the γ-Ga_2O_3 spectra (Figure S5A). Figure S5B applies this estimated scattering correction to the extinction spectrum of Fe_3O_4. Figures S5C and S5D compare the scatter-corrected Fe_3O_4 extinction spectrum to the extinction spectra of the ternary ferrites. These data are consistent with our hypothesis that scattering is a primary contributor to the different magnitudes of the per Fe molar extinction coefficients at energies greater than 2 eV. However, due to the large uncertainty associated with our scattering corrected method, we hesitate to draw any quantitative conclusions from these comparisons.

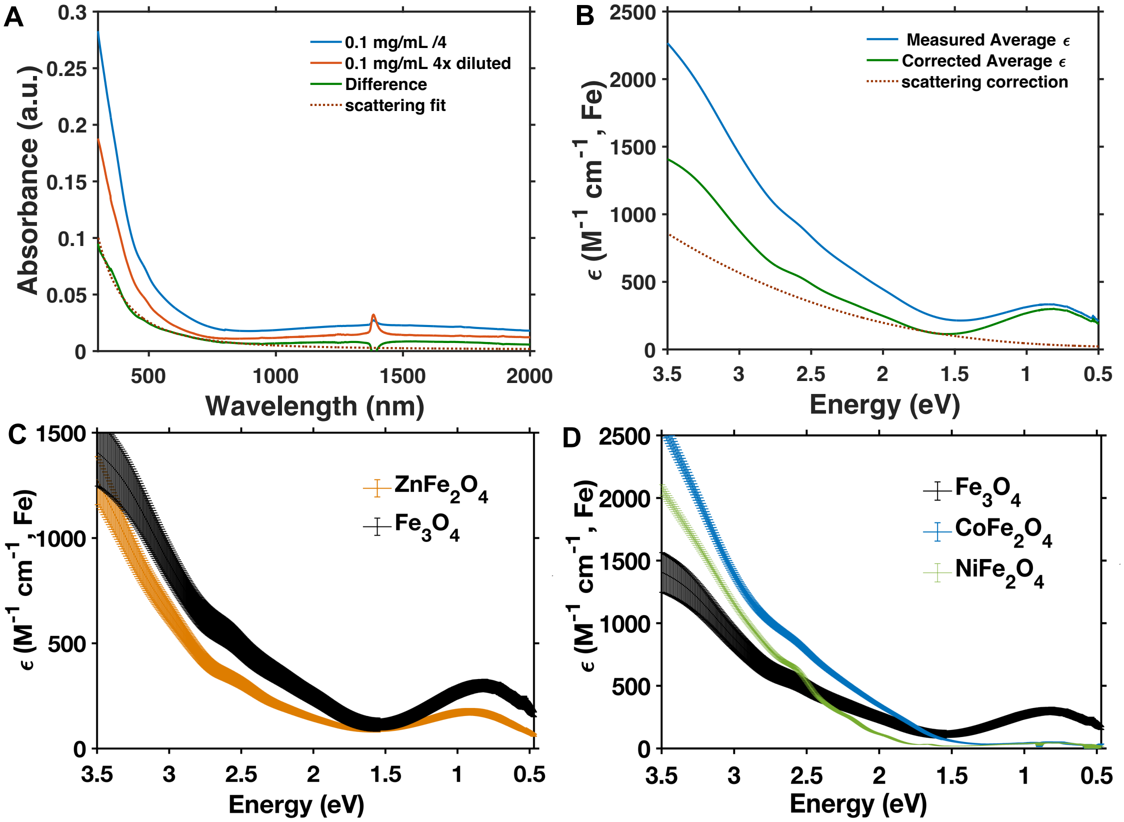

Figure S5. A) Plot of the absorption spectra of a 0.1 mg/mL sample of Fe_3O_4 in tetrachloroethylene divided by four (blue) and a 0.1 mg/mL sample diluted by a factor of four (orange). The difference of these two spectra is plotted in dark green and fit to equation S3 (dashed dark red line). The fit parameters are $k = 7.1 \times 10^5$, $n = 2.766$, $b = 0.0014$. **B)** Plot of the measured (blue) and corrected (green) average per Fe molar extinction spectra of Fe_3O_4. The dashed red line plots the scattering background obtained from the fit conducted in part A in per molar extinction units. **C)** Scatter-corrected per Fe molar extinction spectrum of Fe_3O_4 (black) plotted with the per Fe molar extinction spectrum of $ZnFe_2O_4$ (orange). **D)** Scatter-corrected per Fe molar extinction spectrum of Fe_3O_4 plotted with the per Fe molar extinction spectra of $CoFe_2O_4$ and $NiFe_2O_4$.

Linear Response Hubbard and Hund Parameters

Converged values for plane wave cutoff and k-point grid were determined using the 14-atom primitive cells of the spinel oxide materials. To calculate the Hubbard *U* (electron correlation correction)[3] and Hund *J* (local magnetization correction)[4] for the open-shell metal ions in the ferrite and gallate materials, a linear response to external perturbation method was used.[3,5] In many

Hubbard-corrected computations, a U_{eff} parameter ($U_{eff} = U - J$) is implemented, which combines the electron correlation and local magnetization corrections into a single parameter.[6] The benefit of separately calculating the *U* and *J* parameters from a linear response method is that it provides an intrinsic value that has been shown to provide accurate electronic and vibrational results.[5,7] However, since magnetization is not variational with the total energy of the system, the linear response approach to computing an explicit J value can only be applied to systems where the ground state has no net magnetic moment.[5] To determine intrinsic Hubbard and Hund parameter as accurately as possible, an antiferromagnetically aligned ferrite is used in the linear response method for the materials in this study with a non-zero net magnetization (*vide infra*), and the ferrimagnetic alignment is then imposed for the final electronic calculations. This method was used to calculate the Hubbard and Hund corrections for T_d Co^{2+} and O_h Fe^{3+} in $CoFe_2O_4$, and T_d Fe^{3+}, O_h Fe^{3+} and O_h Ni^{2+} in fully inverted $NiFe_2O_4$. In the case of materials that contain paramagnetic metal centers but have a ground state with no net magnetization ($ZnFe_2O_4$, $CoGa_2O_4$ and $NiGa_2O_4$), the antiferromagnetic spin alignment of paramagnetic ions was maintained from the linear response method to the final calculation of electronic states. The Hubbard and Hund corrections were determined for O_h Fe^{3+} in $ZnFe_2O_4$, T_d Co^{2+} in $CoGa_2O_4$ and O_h Ni^{2+} in fully inverted $NiGa_2O_4$. Because there are no open-shell *d* metals in $ZnGa_2O_4$, this material was calculated with no Hubbard or Hund corrections. The electronic bands and projected densities of states were determined by first calculating the self-consistent field (SCF) wavefunctions with associated Hubbard and Hund corrections and then performing a Fourier interpolation to a larger grid of k-points. Table S4 lists the Hubbard and Hund parameters calculated for the various ternary spinel oxide materials using these methods.

Table S4. Hubbard and Hund parameters for ferrite DFT calculations determined from linear response methods.

**Ferrite Spinel Oxides**		**Gallate Spinel Oxides**	
**$CoFe_2O_4$**		**$CoGa_2O_4$**	
T_d Co^{2+}	U: 2.84388<br>J: 1.35399	T_d Co^{2+}	U: 2.84388<br>J: 1.35399
O_h Fe^{3+}	U: 3.42907		

	J: 3.20380		
**$NiFe_2O_4$**		**$NiGa_2O_4$**	
O_h Ni^{2+}	U: 3.90927<br>J: 1.05339	O_h Ni^{2+}	U: 3.66924<br>J: 1.87018
O_h Fe^{3+}	U: 3.35521<br>J: 2.55839		
T_d Fe^{2+}	U: 3.24468<br>J: 3.08658		
**$ZnFe_2O_4$**			
O_h Fe^{3+}	U: 3.02659<br>J: 3.11699		

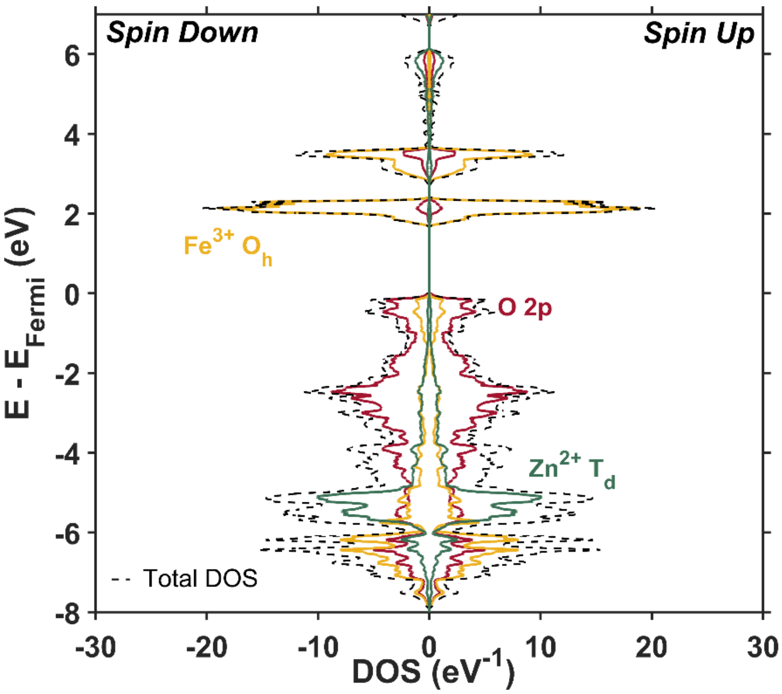

Figure S6. Spin-resolved projected density of states of $ZnFe_2O_4$, calculated with DFT+*U*+*J*.

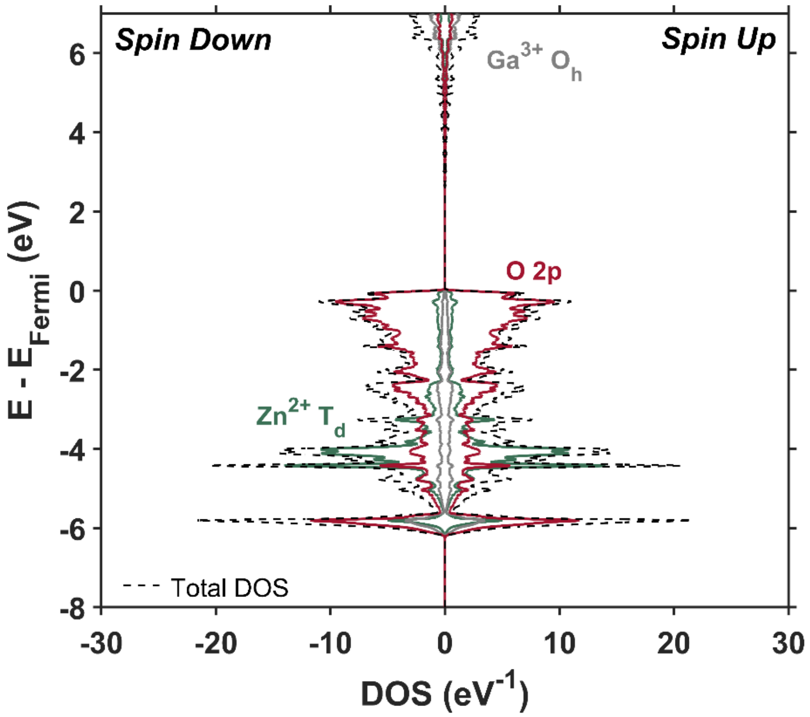

Figure S7. Spin-resolved projected density of states of $ZnGa_2O_4$, calculated with DFT+*U*+*J*.

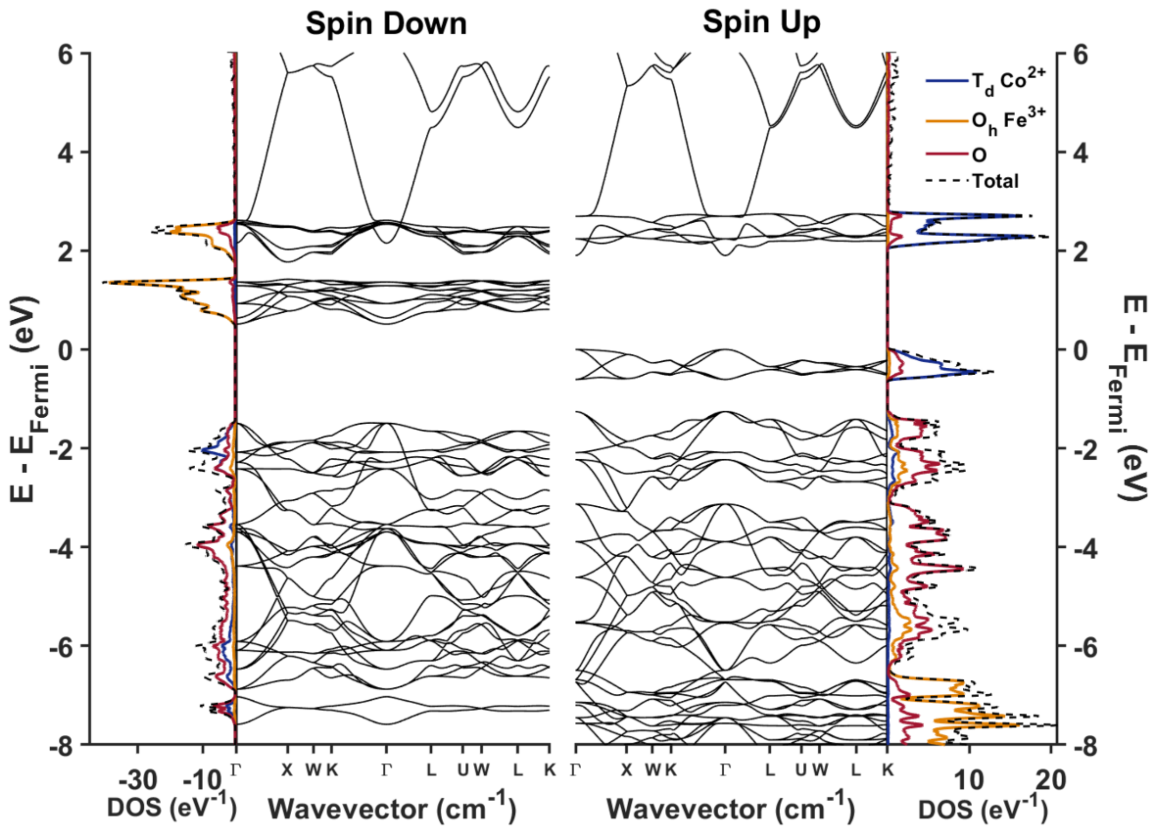

Figure S8. Spin-resolved band structure and associated projected density of states of normal $CoFe_2O_4$, calculated with DFT+U+J.

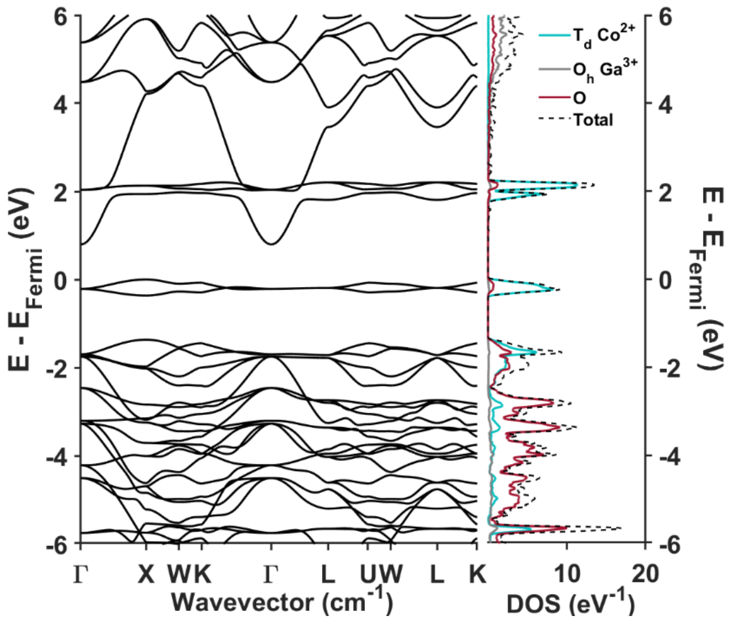

Figure S9. Band structure and associated projected density of states (complex conjugate of DOS plotted in Figure 5B, main text) of normal $CoGa_2O_4$, calculated with DFT+*U*+*J*.

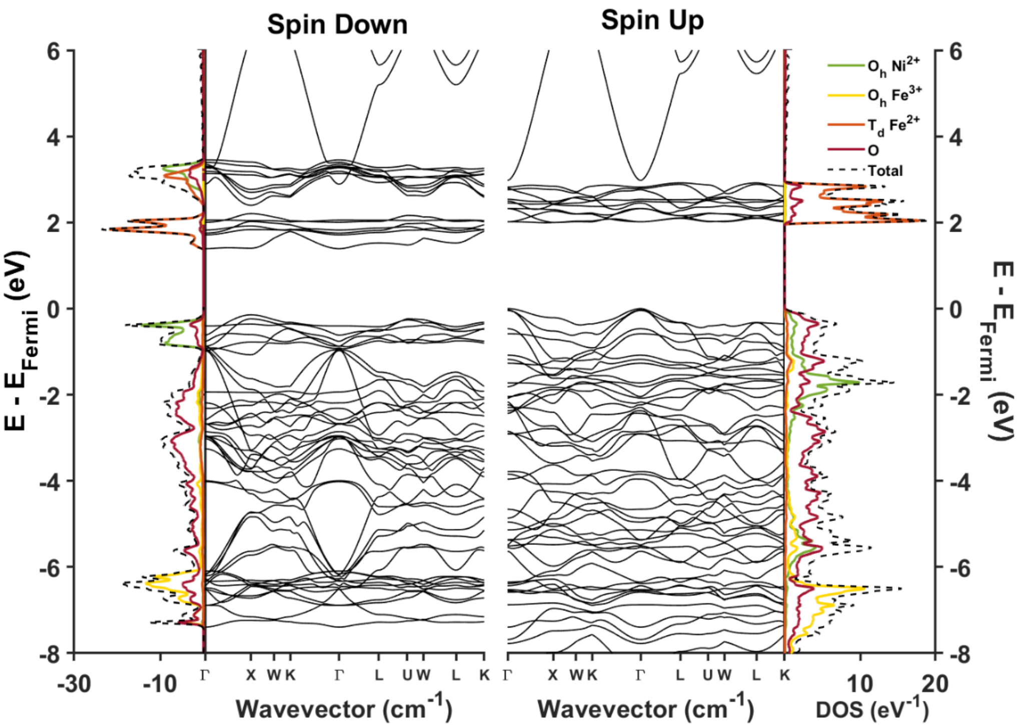

Figure S10. Spin-resolved bands structure and associated projected density of states of fully inverted $NiFe_2O_4$, calculated with DFT+*U*+*J*.

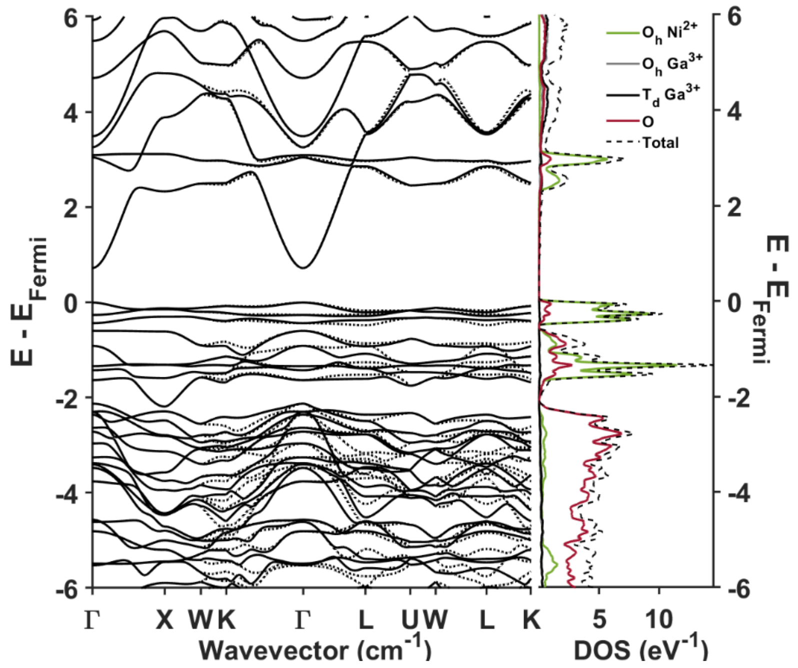

Figure S11. Spin-resolved bands structure and associated projected density of states (complex conjugate of Figure 5D, main text) of fully inverted $NiGa_2O_4$, calculated with DFT+U+J. The solid bands are spin up, while the dotted bands are spin down.

References

(1) Pilkington, T. J.; Bignucolo, M. W.; Ainsworth, J. P.; Siemann, S. An Excel Fitting Routine for Correcting Protein Absorption Spectra for Scatter. *Anal. Biochem.* **2023**, *678*, 115269.

(2) Basalla, A. J.; Kendrick, B. S. Correcting Ultraviolet-Visible Spectra for Baseline Artifacts. *J Pharm Sci.* **2023**, *112* , 3240–3247.

(3) Cococcioni, M.; De Gironcoli, S. Linear Response Approach to the Calculation of the Effective Interaction Parameters in the LDA + U Method. *Phys. Rev. B* **2005**, *71*, 035105.

(4) Himmetoglu, B.; Wentzcovitch, R. M.; Cococcioni, M. First-Principles Study of Electronic and Structural Properties of CuO. *Phys. Rev. B* **2011**, *84*, 115108.

(5) Shelton, J. L.; Knowles, K. E. Polaronic Optical Transitions in Hematite (α-Fe_2O_3) Revealed by First-Principles Electron–Phonon Coupling. *J Chem Phys.***2022**, *157*, 174703.

(6) Cococcioni, M.; De Gironcoli, S. Linear Response Approach to the Calculation of the Effective Interaction Parameters in the LDA + U Method. *Phys. Rev. B* **2005**, *71*, 035105.

(7) Himmetoglu, B.; Floris, A.; De Gironcoli, S.; Cococcioni, M. Hubbard-Corrected DFT Energy Functionals: The LDA+U Description of Correlated Systems. *Int. J. Quantum Chem.* **2014**, *114*, 14–49.
